# Not a Sip: Effects of Zero Tolerance Laws on Road Traffic Fatalities

**DOI:** 10.1002/hec.70056

**Published:** 2025-11-24

**Authors:** Andres Ramasco

**Affiliations:** ^1^ Department of Economics University of Notre Dame Notre Dame Indiana USA

**Keywords:** drinking behavior, drunk driving, preventable deaths, Traffic fatalities

## Abstract

A substantial proportion of alcohol related fatalities and their consequences are preventable, prompting policymakers to implement measures aimed at reducing these deaths. I exploit time and geographic variation in the adoption of zero‐tolerance laws in a difference‐in‐differences design to study the impact of these regulations on traffic‐related incidents. Using county‐level data, I find no sizable reductions in fatalities and an increase in injury counts after the adoption of such laws. I do not find significant changes in several measures of alcohol consumption, consistent with the lack of reduction in driving fatalities.

## 1 Introduction

With approximately 1.2 million deaths each year, Road Traffic Fatalities (RTF) remain a leading cause of preventable death worldwide, and governments have responded with a variety of road safety regulations. Among the most prominent are laws restricting alcohol consumption while driving, yet evidence on their effectiveness has been mixed (Carpenter 2004; Francesconi and James 2021; Guimarães and da Silva 2019).

One common regulation limits the maximum legal blood alcohol concentration (BAC). This regulation increases the non‐monetary costs of drinking and driving, and potentially deters this risky behavior (Evans et al. 1991; Hansen 2015; Kenkel 1993). The extreme version of this regulation, known as a Zero Tolerance Law (ZTL), sets the allowable BAC to zero. While alcohol impairs driving, the relationship between BAC and accident risk is exponential, with both the probability and severity of crashes concentrated among drivers with very high BAC levels, as shown by (M. T. Compton et al. 2009). Given this, both theoretical and empirical papers such as Grant and Lewis 2014 and Grant, 2010 argue that ZTLs often fail to reduce traffic fatalities and may even have unintended consequences. By targeting moderate drinkers rather than heavy ones, such laws can reduce the incentive to drink responsibly and do not substantially discourage binge and heavy drinking, as discussed by Kenkel 1993; Hansen 2015.

In this paper, I study the impact of ZTLs on traffic deaths and injuries in Argentina.^1^ To do so, I begin by collecting information on ZTL adoption from official government bulletins and legislative digests published by each state and county between the years 2013 and 2022.^2^ For my primary outcomes, road traffic injuries and fatalities, I exploit regional and temporal variation in a difference‐in‐differences framework. To avoid biases introduced by the staggered treatment timing, I implement the estimator proposed by Callaway and Sant’Anna 2021. To provide evidence on the mechanisms behind my results, I conduct additional analyses on the impacts of ZTLs on alcohol consumption, drunk driving, and other health outcomes.^3^

I find that ZTLs lead to a significant and persistent increase in traffic‐related injuries—ranging from 5.3% to 6.7%, depending on the specification—and have no detectable effect on traffic fatalities up to 5 years after their adoption. The event‐study estimates show a clear upward shift in injuries following implementation and a potential increase in fatalities, although the standard errors are too large to draw definitive conclusions. However, I can rule out any reduction in fatalities greater than 6%. Thus, the empirical evidence suggests that the policy backfired and did not achieve its intended goals. Together, these findings contrast with previous results in the literature on BAC regulations focused on young drivers (Benson et al. 1999) or minimum legal drinking age laws (Dee 1999), and contribute new evidence for the broader adult population.

Three mechanisms rationalize my findings. First, I analyze behavioral changes in the subset of states for which I observe self‐assessed measures of alcohol consumption and drunk driving. While I find relatively small decreases in binge drinking, this does not translate into meaningful changes in drunk driving or average alcohol consumption. Second, because setting discrete legal limits on alcohol consumption may induce bunching behavior at the limits, I examine whether the probability of consuming more than two drinks—an amount approximating moderate consumption near the prior legal threshold—increases after the reform. The results confirm this hypothesis: I find a significant increase in the likelihood of moderate drinking, consistent with the idea that lowering the legal BAC limit reduces the marginal cost of additional consumption for individuals who previously clustered around the 0.05 BAC threshold. Third, I test whether the reform impacts alcohol‐related hospitalizations–a proxy for severe alcohol impairment–and find no sizable changes. These results support the hypothesis that the increase in crash‐related injuries, in the absence of a rise in fatalities, may be driven by upward shifts in moderate drinking.

My contribution is twofold. First, this paper contributes to the literature on drunk‐driving policies by providing new evidence on the effects of ZTLs for the entire adult population. While previous studies have focused on policies targeted to young drivers (Dee 1999) and ZTLs for under 21 drivers (Carpenter 2004; Liang and Huang 2008), this study analyzes behavioral responses across all age groups. By doing so, it sheds light on whether stricter drunk‐driving regulations influence the broader adult population, a question that has received limited empirical attention to date.

Second, this paper offers new evidence on the effects of ZTLs in a large, decentralized, middle‐income country—Argentina—where staggered adoption across states creates a natural setting for causal inference. In contrast to prior studies in Chile (Otero and Rau 2017), Uruguay (Davenport et al. 2021), and Brazil (Guimarães and da Silva 2019), the combination of variation in policy timing and decentralized enforcement allows this paper to deliver more granular evidence on medium‐ and long‐run effects. This complements a growing literature on drunk‐driving policies in high‐income countries, including the U.S. and Western Europe (Carpenter and Dobkin 2009; Grant, 2010, 2016; Liang and Huang 2008; Norström and Laurell 1997). Importantly, this paper also addresses a common challenge in studies using granular geography—namely, the potential for spillovers across adjacent areas (Francesconi and James 2021). Overall, the study complements the existing evidence in high‐income countries by providing a more robust assessment of the effectiveness of ZTLs in a developing‐country context.

## 2 Background

### 2.1 Legal Background

In 2013, before the first ZTL was implemented, Argentina's national Road Traffic Fatality (RTF) rate stood at 13.6 per 100,000 people. This figure was slightly lower than the regional average for the Americas but remained quite high by global standards.

ZTLs are a strict version of one of the policies implemented to reduce traffic accident fatalities, namely drunk driving regulations. Although zero‐tolerance laws have been analyzed in previous studies, they are mostly confined to the United States, particularly regarding individuals under 21. The policy in Argentina differs in two substantial ways. First, the target population is the entire adult population, which is not a trivial distinction, as individuals from different age groups are expected to demonstrate varying propensities for risky behaviors as well as different attitudes toward alcohol consumption. Second, in the United States, these laws are enforced alongside the prohibition of acquiring and consuming alcoholic beverages, as the legal minimum age for this is also 21 years old. In the case of the law applied in Argentina, the specific effect of the law on drunk driving can be isolated without being confounded by this prohibition on alcohol consumption.

Argentina is a federally organized country with 23 states and an autonomous capital city. Each state is subdivided into departments or partidos, which I refer to as counties. While national traffic laws provide a general framework, enforcement and implementation are somewhat decentralized, with each state and municipality adapting regulations to their local contexts. This decentralization is evident in the staggered adoption of Zero Tolerance Laws across states, as seen in Table 1, where some provinces implemented the policy as early as 2014, while others only did so in 2022. It is also worth noting that some counties deviate from their state guidelines, by enforcing stricter or more lenient policies, as shown in Figure A7.^4^

**TABLE 1:** Provinces adopting zero tolerance by year.

Year	Provinces adopting zero tolerance	Combined population	Cumulative population share (%)
2014	Córdoba	3,518,663	7.7%
2015	Salta	1,285,600	10.5%
2016	Misiones, Neuquén, Tucumán	3,289,456	17.8%
2018	Entre Ríos, Santa Cruz	1,588,981	21.3%
2019	Jujuy, Tierra del Fuego	858,098	23.1%
2020	Río Negro	677,388	24.6%
2021	Santa Fe	3,310,908	31.9%
2022	Buenos Aires, Chaco, Chubut, La Rioja	18,210,488	71.9%

Before 1994, alcohol consumption while driving in Argentina was regulated arbitrarily. The protocols for handling drunk driving were not standardized, and they varied significantly between states due to the autonomy of local police forces. Even major cities had their own municipal guards, which further contributed to inconsistent enforcement. There were no clear, documented guidelines for penalties, and the measures taken often relied on the subjective judgment of traffic officers or police.

The situation around drunk driving enforcement changed substantially with the enactment of Traffic Law 24,449 in 1994, which introduced more standardized regulations for alcohol‐related offenses, establishing specific penalties for driving under the influence. It also marked the beginning of a shift toward more objective enforcement measures, such as the use of electronic breathalyzers, rather than relying solely on the discretion of officers. Furthermore, the law set the legal limit for blood alcohol at 0.05 g/dL. Prior to the Zero Tolerance Law (ZTL), there was not an established standard to determine impairment.

While the enforcement of particular laws varies across districts, the most standard procedures documented by the National Observatory of Road Safety (ONSV) are random sobriety checkpoints. It is documented that younger individuals, especially those above the minimum driving legal age, are, on average, more prone to be involved in risky driving behavior (Huh and Reif 2021; Zador 1991).

Although penalties for drunk driving vary by jurisdiction, they are generally not severe for first‐time offenders, with the main consequences being vehicle impoundment and a fine.^5^ For repeat offenders, depending on the jurisdiction, their license may be revoked; however, this is not typically the case. Data from the National System of Penitentiary Statistics (SNEEP) shows that the vast majority of offenses leading to incarceration are unrelated to drunk driving or fatal car accidents. The most recent SNEEP report (2021) indicates that fewer than 1% of incarcerated individuals are associated with these causes.

Even though it is common to see awareness campaigns about drunk driving throughout Argentina, there is no clear evidence indicating that districts implementing zero‐alcohol laws tend to make greater use of these strategies. It is common to see on national television government campaigns addressing the effects of drinking and driving. Some provincial governments, such as those of the Autonomous City of Buenos Aires, the state of Buenos Aires, and Entre Ríos, also carry out awareness campaigns for both residents and tourists. However, these campaigns have been in place since before the implementation of zero‐alcohol laws, meaning there is no clear correlation between the introduction of ZTL and an increased emphasis on such campaigns.

### 2.2 Conceptual Background

When the legal BAC limit is lowered from 0.05 to 0.00, standard economic theory predicts a shift in the distribution of BAC levels among drivers. Specifically, we expect fewer drivers with BAC levels between 0 and 0.05, and increases in the mass at zero and above 0.05 (Grant and Lewis 2014; Kenkel 1993). This pattern arises because the reform alters the marginal cost of drinking in a non‐uniform way: it increases the cost for individuals who previously remained within the legal range (i.e., those near 0.05), while effectively reducing it for those who would have already exceeded that threshold. Since all positive BAC levels are now penalized equally, moderate drinkers no longer have an incentive to limit consumption just below the prior legal limit.

These behavioral responses are well documented in the literature. Kenkel (1993) argues that overly stringent driving regulations may induce compensatory behaviors that undermine the intended benefits of the policy. Similarly, Evans et al. (1991) and Hansen (2015) show that individuals with high BAC levels are particularly difficult to deter through regulation, while moderate drinkers are more responsive to marginal policy changes.

It is well established that the relationship between BAC and accident risk is highly non‐linear. Theoretical models suggest this relationship is exponential (Grant and Lewis 2014), with particularly low accident risk at BAC levels below 0.05. Empirical evidence confirms that drivers involved in fatal crashes typically have BAC levels well above legal thresholds (M. T. Compton et al. 2009; Zador 1991).

Taken together, these findings suggest that the effectiveness of drunk‐driving policies hinges on their ability to deter heavy drinkers. Since most alcohol‐related traffic deaths involve drivers with BACs substantially above 0.05, lowering the legal limit may have limited safety benefits—particularly if it deters moderate drinkers more than high‐risk individuals. In such cases, Zero Tolerance Laws (ZTLs) could generate unintended consequences by discouraging compliance or increasing consumption among marginal drinkers (Hansen 2015; Kenkel 1993). This concern is especially relevant in Argentina, where the pre‐reform threshold was already low.

## 3 Data

### 3.1 Treatment Status

I assemble a dataset on the implementation of ZTLs for each state and county, using administrative data from state and municipality legislative digests and official government bulletins.^6^ In cases where I estimate a model at the state level, I assign a state to treatment if more than 60% of its population is affected by a ZTL.

### 3.2 Health Outcomes

My main outcome variables are road traffic deaths and injuries. I use four administrative datasets to quantify the effect of ZTLs on health outcomes. First, I use administrative data provided by the National System of Criminal Information (SNIC), which is dependent on Argentina's Ministry of Security. These data include information on the number of crimes and victims for 10 broadly defined categories, including road traffic accidents. The SNIC is a system to collect and consolidate data across law enforcement agencies, including provincial and federal police forces. The information collected stems from the Early Warning System (SAT), a procedure implemented by the Ministry of Security to collect detailed information on four types of crime: property crimes, murders, suicides, and traffic fatalities. For this paper, I focus on this latter module of the SNIC. The granularity of this data allows me to make county‐to‐county comparisons, which reduces aggregation bias and increases statistical power relative to state‐level analyses. This database reports annual counts of fatalities and injuries for the 2014–2022 period.

Second, I use vital statistics from the Argentinian Ministry of Health (MS), which annually provides counts of death by cause following the ICD‐10 classification at the state level. Each database register comes from death certificates completed by doctors covering 2005–2021. Third, I use data from the ONSV, since official vital statistics tend to underestimate the true number of traffic fatalities when compared with hospital registry procedures. ^7^ 8 These data provide counts of fatal accidents and victims monthly at the state level for 2015–2021. It provides more granularity and statistical power than the MS data since this dataset provides counts for the outcome variables at higher frequency (monthly rather than annually) for almost the same number of periods. One limitation of the data is that it begins after certain states had already adopted ZTLs. A common limitation of these datasets is that I cannot distinguish alcohol‐related from non‐alcohol‐related fatalities.

**FIGURE 1:**
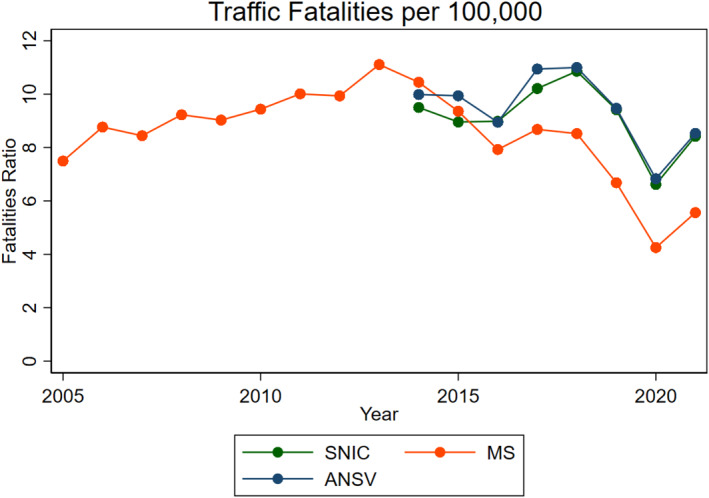
Road traffic fatalities across time. This plot presents the fatalities rate from three different data sources: National System of Criminal Information (SNIC) reports, vital statistics from the Ministry of Health of Argentina (MS), and fatality counts from the National Road Safety Agency (ANSV).

Figure 1 compares traffic fatality rates across alternative data sources over time. The figure reports average deaths per 100,000 people and reveals consistent patterns across the three series. It also highlights systematic underreporting in the MS dataset, likely due to missing information in the completion of death certificates.

### 3.3 Behavioral Outcomes

I use two different surveys to assess the impact of ZTLs on people's behavior. First, I use the National Survey of Risk Factors (ENFR), a nationally representative household survey featuring self‐assessed information on substance use, such as alcohol consumption, and impaired driving. It is composed of two cross‐sections (2013 and 2018).

Last, I use hospital discharge data from the Department of Health Information and Statistics (DEIS), an office within the MS. This dataset provides annual counts of discharges by gender and cause at the state level. To capture outcomes more directly related to excessive alcohol consumption, I focus on discharges associated with alcohol‐related disorders, specifically cases of alcohol poisoning.

### 3.4 Labor Market Variables

To control for possible confounding factors, I calculate unemployment and private‐sector employment from the Permanent Households Survey, a rotating panel that interviews households every quarter.^9^ This survey is representative of approximately 80% of the population since it covers most urban areas in the country, which has a considerable urban population (92%). In particular, I merge the four quarters for 2013 and use them as my pretreatment period.

### 3.5 Descriptive Statistics

Table 2 shows the means for several pretreatment characteristics associated with the outcome variables across the treatment and control states.^10^ Table 2 also shows the means and differences of sociodemographic variables across the treatment and control groups. No differences are observed except the slightly higher concentration of young adults among the treated units, although the magnitude of the difference is not particularly large. Altogether this balance table provide evidence of no significant differences in the treated units, alleviating concerns about treatment endogeneity. Importantly, there are no statistically significant differences between the treatment and control groups on road traffic fatalities or injuries, my main outcome variables.

**TABLE 2:** Balance table.

	Control		Treatment		
	Mean	SD	Mean	SD	Diff
County‐level variables					
Fatalities rate	13.35	10.53	13.17	7.52	0.18
RT injuries rate	189.17	54.13	294.41	224.64	−105.24
Observations	1704		2853		
State‐level variables					
Age < 18	0.31	0.02	0.30	0.05	0.01
28 ≥ age ≥ 19	0.19	0.01	0.20	0.01	0.00
66 ≥ age ≥29	0.4	0.02	0.39	0.02	−0.00
Age ≥ 65	0.09	0.01	0.08	0.03	−0.01
Educ > HS	0.44	0.11	0.52	0.15	−0.08
Income per capita	2.905	0.599	3.245	1.214	−0.339
Unemployed	0.07	0.02	0.05	0.02	0.01
Private Emp	0.80	0.06	0.80	0.06	0.01
Cars per capita	0.32	0.25	0.25	0.13	0.07
Observations	11		13		

*Note:* Treatment indicates treatment at the state level up until 2021. Rates are the frequency per 100,000 people. Income and employment variables are from the Permanent Household Survey. Income is expressed in thousand of pesos.

Although my main specification is based on county‐level variables, sociodemographic data at that geographical level are not available; therefore, I use state‐level data to show balance. Nevertheless, there are no serious concerns about within‐state heterogeneity in these variables that might mask differences across the treated and nontreated counties.

For my main specification, in which I use the data from SNIC, the observation unit is a county–year, with the sample period encompassing 2014–2022. Since I only have access to state‐level data for hospital discharges and the heterogeneity analysis, the unit of observation for those analyses is the state‐year rather than the county‐year.

## 4 Empirical Strategy

I estimate the impact of ZTLs on traffic health outcomes within a differences‐in‐differences framework, comparing treated to nontreated units before and after ZTL implementation. A recent literature has documented the risks involved when using two‐way fixed effects (TWFE) estimators in the presence of staggered rollouts Callaway and Sant’Anna (2021); De Chaisemartin and d’Haultfoeuille (2022); Goodman‐Bacon (2021); Sun and Abraham (2021). This literature highlights two key issues: first, the presence of heterogeneous effects across different treated groups, and second, negative weighting. The latter refers to a situation where treated units receive a negative weight when aggregating the estimated effects for each group. This issue can lead to the estimated treatment effect using TWFE having a negative sign even when all individual effects are positive, and vice versa. Therefore, to avoid these issues and allow for treatment heterogeneity across treatment groups, I use the estimator developed by Callaway and Sant’Anna (2021). The first step in the method involves estimating the average treatment effect on the treated (ATT) for group *g* at time *t* (ATT(g,t)).

Let i index units (e.g., counties), t index time (e.g., years or quarters), and g index the first treatment period for each group $G_{g}$.^11^

(1) $$ATT\left(g,t\right)=E\left[Y_{t}-Y_{t-1}|G_{g}=1\right]-E\left[Y_{t}-Y_{t-1}|C=1\right]$$

In the equation above, t=1,…T, and G is the period when a unit first becomes treated. Accordingly, $G_{g}$ is a binary variable equal to one if a unit is first treated in period g, and C is a dummy variable that takes value one for never‐treated observations.

The second step of the Callaway and Sant’Anna (2021) method involves aggregating the previously estimated ATTs, the (g,t)s, in a weighting scheme to make them comparable to standard difference‐in‐difference or event‐study coefficients. In my main specification, I use the following aggregation scheme:

(2) $$\theta_{W}^{O}=\frac{1}{\kappa }\sum_{g\in G}\sum_{t=2}^{T}1\left\{t\geq g\right\}ATT\left(g,t\right)P\left(G=g|G\leq T\right)$$

where $\kappa =\sum_{g\in G}\sum_{t=2}^{T}1\left\{t\geq g\right\}P\left(G=g|G\leq T\right)$. This estimator captures what is called the *overall* treatment effect. An advantage of this parameter is that, unlike the TWFE coefficient, it rules out the possibility of negative weights and potential changes in sign of the estimated effects.

Additionally, to illustrate the dynamic effects of the policy, I run the following event‐study specification:

(3) $$\theta_{es}\left(e\right)=\sum_{g\in \in G}1\left(g+e\leq T\right)P\left(G=g|G+e\leq T\right)ATT\left(g,g+e\right)$$

Last, I use the doubly robust estimator from Callaway and Sant’Anna (2021) to control for state‐varying labor market conditions that might act as confounders. These controls include the unemployment rate, the share of formal workers, and the number of vehicles per capita.

### 4.1 Parallel Trends

Satisfying the parallel trends assumption is a necessary condition for the validity of the empirical strategy. Here, I invoke the assumption of parallel trends concerning never‐treated units. In my setting, a parallel trends violation would mean that the treated counties would have faced different trends from the control units without the treatment. Although this assumption is not directly testable, there exist testable implications related to it. I evaluate these implications in three ways. First, I have already shown above the baseline characteristics of the treated and control units, finding no stark differences. Second, I examine the pretreatment coefficients using the event‐study specification developed by Callaway and Sant’Anna (2021). Third, as a robustness check, I control for state‐level trends in an event study specification to isolate the effect of ZTLs from the effects of confounders.

## 5 Results

### 5.1 Main Estimates

Panel A of Table 3 presents the coefficients on the fatalities rate from Equation (2) as estimated under alternative specifications, together with standard errors clustered at the state level. The estimates in Table 3 indicate an insignificant impact of ZTLs on traffic fatalities. While the estimates lack precision, there is no strong evidence of a meaningful reduction in traffic deaths. Column 1 of Table 3 presents the main specification, showing a positive but not statistically significant coefficient of 0.39, which implies an increase of 3.8% with respect to the mean. Column 2 includes state‐level controls to account for time‐varying potential confounders (number of vehicles per person, unemployment rate, and private employment rate), and as observed, the magnitude and significance of the coefficient are not substantially modified.^12^ Column 3 shows the coefficient that results from my omitting the year 2020 from the estimation to address the concern that the pandemic might have differentially affected the outcome variables across states. The coefficient is slightly larger than that from the main specification in Column 1 but still not statistically significant.

**TABLE 3:** Differences‐in‐differences estimates for health outcomes.

Panel A‐ Dep. Variable: Traffic fatalities			
	(1)	(2)	(3)
Zero tolerance law dummy	0.398	0.326	0.777
	(0.575)	(0.549)	(0.713)
*N*	3746	3746	3306
Mean of Dep. Variable	10.73	13.4	11.05
State controls	N	Y	Y
Excluding 2020	N	N	Y
County FE	Y	Y	Y
Year FE	Y	Y	Y

Panel B‐ Dep. Variable: Traffic injuries			
	**(1)**	**(2)**	**(3)**
Zero tolerance law dummy	75.53***	56.14***	61.80***
	(13.36)	(17.95)	(11.22)
*N*	3746	3746	3306
Mean of Dep. Variable	225.55	246.07	233.32
State controls	N	Y	Y
Excluding 2020	N	N	Y
County FE	Y	Y	Y
Year FE	Y	Y	Y

*Note:* Outcome variables are traffic fatalities and traffic‐related injuries per 100,000 people at the county level. The analysis period is 2014–2022. Standard errors clustered at the state level are reported in parentheses. */**/*** indicates significance at the 10/5/1% levels.

*Source:* Reports from the National System of Criminal Information. County‐level controls include the number of motor vehicles per capita, unemployment and private sector employment.

Panel B of Table 3 shows the results on the rate of traffic‐related injuries reported to the SNIC by local and provincial police forces. A positive effect is observed for all the different specifications, implying that the effect of ZTLs might be the opposite of the one that policymakers expect. Since the coefficients are positive and statistically significant for all specifications, I can reject that the laws cause reductions in injuries.

**FIGURE 2:**
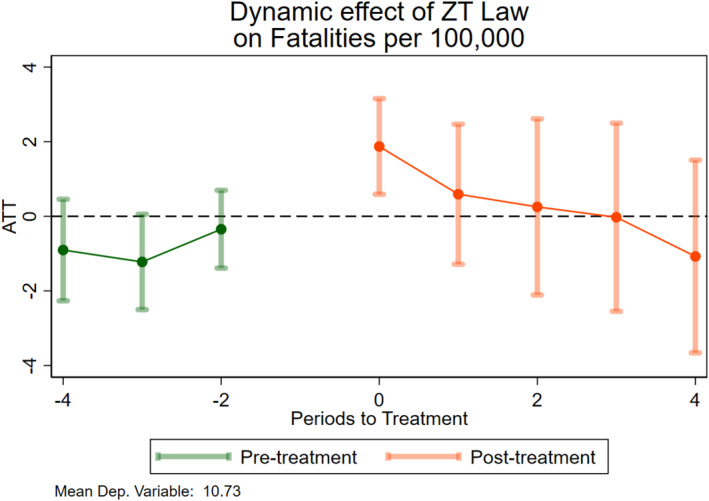
Event study ‐ fatalities. This figure shows point estimates and confidence intervals of the causal effect of ZTLs on the fatalities rate. The base period corresponds to the time when the new policy is passed. Standard errors are clustered at the state level.

Some papers in the literature highlight heterogeneity in the treatment effects across time, that is, how the treatment effects evolve at different periods after treatment (Carpenter and Dobkin 2009). For example, Otero and Rau (2017) document a sharp decrease in drunk driving in the months right after implementation of a new law that subsequently vanishes. To assess the dynamic effects of the laws through time, I run an event‐study specification on the outcomes of interest in the county‐level data from SNIC. Figure 2 shows the event‐study coefficients from Equation (3) for the fatalities rate. The laws have no sizable effect on traffic fatalities per 100,000 people. The coefficient for the first period after treatment is positive and the only one showing statistical significance, denoting an *increase* in fatalities after the implementation of ZTLs, followed by modest but insignificant increases. I can observe a decreasing trend, although I do not have statistical power to rule out effects different from zero. Nevertheless, as noted in Panel A of Table 3, the overall treatment effect is not statistically significant. Although the standard errors are relatively imprecisely estimated, I can rule out a reduction of a magnitude larger than 6% in average.

**FIGURE 3:**
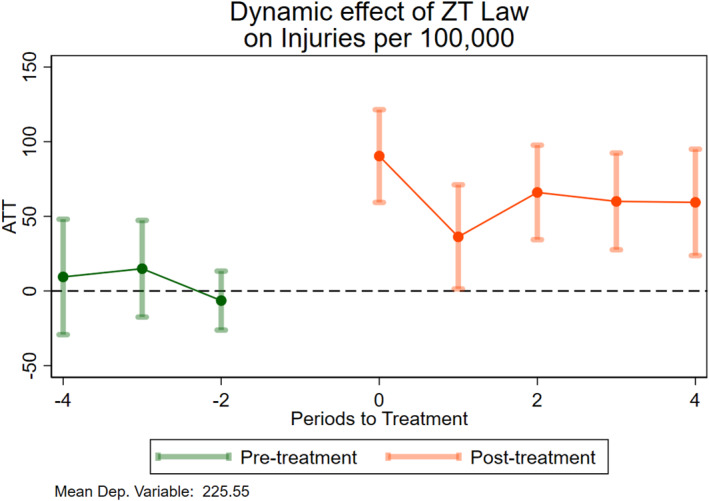
Event study: Traffic injuries. This figure shows point estimates and confidence intervals of the causal effect of ZTLs on the injury rate. The base period corresponds to the time when the new policy is passed. Standard errors are clustered at the state level.

Similarly, in Figure 3, one can observe the dynamic response of traffic‐related injuries per 100,000 at the county level. The estimates show positive coefficients, indicating *increased* traffic injuries. I observe an abrupt jump in period zero, followed by a decrease in period one, although this coefficient is positive and statistically significant, implying an abrupt increase in injuries followed by small increases afterward. Overall, I see a clear and steady increase in injuries. The coefficients for the preintervention periods in both figures provide suggestive evidence that the parallel trends assumption holds.

## 6 Mechanisms

The previous section showed that ZTLs increase traffic injuries without reducing fatalities. This section explores behavioral mechanisms that could explain these results. As discussed in Section 2.2, lowering the legal BAC limit from 0.05 to 0.00 may reduce the incentive to moderate consumption. Therefore, among those who drink, we expect higher intensity of alcohol use.

Why do I find these *unexpected* results on fatalities and injuries after the reforms? Is the population modifying its drinking behavior—i.e., does the BAC distribution change? Previous articles show mixed evidence on whether reducing the maximum BAC to zero can generate sizable decreases in fatalities (Norström and Laurell 1997). More recent studies point out that the elasticity of the supply of offenses with respect to the probability of conviction on the left tail of the distribution is reasonably low, given the relatively low increased relative risk (R. P. Compton et al. 2002), so the potential for sizable reductions in fatal crashes is small. In particular, papers on optimal drunk driving policies point out the pitfalls of more restrictive laws such as ZTLs (Grant and Lewis 2014; Liang and Huang 2008).

To evaluate these mechanisms, I analyze different behavioral outcomes related to traffic fatalities and injuries using self‐reported measures from the Risk Factor Survey (ENFR): drinking in the last month, drinking habit, binge drinking, and drunk driving. *Binge Drinking* equals one if the respondent had five or more drinks on one occasion in the last 30 days. *Drinking habit* follows INDEC (National Institute of Statistics and Census) guidelines: more than two drinks per day for men and more than one for women, conditional on having consumed alcohol in the past month.

Given that I rely on two ENFR cross‐sections, I estimate a standard difference‐in‐differences probit model:

(4) $$P\left(Y_{\mathrm{ist}}=1|X_{\mathrm{ist}}\right)=\Phi \left(\beta_{0}+\beta_{1}\cdot {\text{Post}}_{t}+\beta_{2}\cdot {\text{Treated}}_{s}+\beta_{3}\cdot \left({\text{Treated}}_{s}\times {\text{Post}}_{t}\right)+\gamma^{\prime }X_{it}+\delta^{\prime }X_{s}\right)$$

where $Y_{\mathrm{ist}}$ is the outcome of interest, $Post_{t}$ indicates post‐policy observations (2018), $Treated_{s}$ equals one for treated states, $X_{it}$ and $X_{s}$ are control vectors, and Φ is the standard normal CDF. Linear probability model results appear in Appendix Table A5 and yield similar conclusions.^13^

Table 4 reports the average marginal effects of ZTLs on self‐reported behaviors. Most outcomes, including drunk driving, show no statistically significant changes, with the exception of a 6.4 pp decline (28% relative to the mean) in binge drinking (Column 3).

**TABLE 4:** Difference‐in‐differences estimates of ZTL effects on behavioral outcomes.

	(1)	(2)	(3)	(4)
	Drinking	Drinking	Binge	Drunk
		habit	drinking	driving
Zero tolerance x POST	0.0127	−0.00815	−0.0643^a^	−0.0139
	(0.0280)	(0.0159)	(0.0255)	(0.0221)
Weighted	Yes	Yes	Yes	Yes
State FE	Yes	Yes	Yes	Yes
Year FE	Yes	Yes	Yes	Yes
Observations	49,175	30,389	30,644	26,033
Mean of Dep. Variable	0.654	0.153	0.222	0.1373

*Note:* Standard errors clustered at the state level in parentheses. Dependent variables are binary and take value 1 when the interviewed individual answers affirmatively to the questions. Coefficients reported are average marginal effects from a probit model. Column 1 includes observations for the entire sample who responded to the survey. Columns 2 and three exclude observations for individuals who do not drink and differ only in their non‐response rate. Column 4 excludes observations for individuals who do not drive on a regular basis.

*Source:* National Risk Factor Survey.

*p<0.10.

***p<0.01.

^a^ p<0.05.

Because crash risk is non‐linearly related to BAC (R. P. Compton et al. 2002; Ruhm 1996), I next examine intensive margin effects among those who drank in the last month. Table 5 shows that the probability of consuming more than two drinks increases post‐ZTL, particularly among moderate drinkers (Column 2). This suggests that the marginal cost of additional drinks fell once any positive BAC carried the same penalty.

**TABLE 5:** Difference‐in‐differences estimates of ZTL effects on probability of consuming more than two drinks.

	(1)	(2)
	+2 drinks	+2 drinks
Treated	0.0258^b^	0.0316^a^
	(0.0128)	(0.0179)
Year FE	Yes	Yes
State FE	Yes	Yes
Observations	29,167	20,760
Mean of Dep. Variable	0.801	0.717

*Note:* Standard errors in parentheses. Standard errors clustered at the state level in parentheses. The dependent variable equals 1 for individuals who drink more than two drinks Coefficients reported are average marginal effects from a LPM. Column 1 includes observation for the entire sample who reported having at least one drink. Column 2 excludes individuals who reported drinking more than eight drinks.

*Source:* National Risk Factor Survey.

***p<0.01.

^a^ p<0.10.

^b^ p<0.05.

Taken together, these results suggest that ZTLs reduced binge drinking but increased moderate drinking, consistent with the observed pattern of more injuries but no reduction in fatalities. Prior research indicates that BAC levels below 0.10 are more strongly associated with non‐fatal rather than fatal crashes (R. P. Compton et al. 2015).

To complement these self‐reported outcomes, I use administrative data on hospital discharges for alcohol poisoning. Table 6 shows a positive but statistically insignificant impact overall and across age groups. The pattern is consistent with unchanged behavior among heavier drinkers, who are key contributors to traffic fatalities (R. P. Compton et al. 2002; Sloan et al. 1995).

**TABLE 6:** Difference‐in‐differences estimates for hospital discharges related to alcoholism.

		Age			
	Full	15–24	25–34	35–44	44 +
	sample				
Treated	6.09	6.06	4.65	6.04	10.34
	(8.92)	(13.86)	(7.6)	(13.19)	(12.79)
Weighted	Yes	Yes	Yes	Yes	Yes
Province FE	Yes	Yes	Yes	Yes	Yes
Year FE	Yes	Yes	Yes	Yes	Yes
Observations	392	392	392	392	392
Mean of Dep. Variable	30.10	33.74	21.63	28.49	32.11

*Note:* Standard errors clustered at the state–gender level in parentheses. The outcome variable is hospital discharges due to alcohol consumption per 100,000 people. Data from the City of Buenos Aires and Santiago del Estero are not available.

*p<0.10.

**p<0.05.

***p<0.01.

### 6.1 Robustness

#### 6.1.1 Alternative Data Sources

To address concerns about underreporting from state police sources, I re‐estimate the model using data from the Ministry of Health and the National Road Safety Agency. Appendix Table A1 confirms the robustness of the main estimates.

#### 6.1.2 Urban Areas

Figures A3, A4, A5, A6 show similar dynamic effects in urban and rural areas, indicating no substantial heterogeneity along this dimension.

#### 6.1.3 Leave‐One‐Out Estimates

**FIGURE A1:**
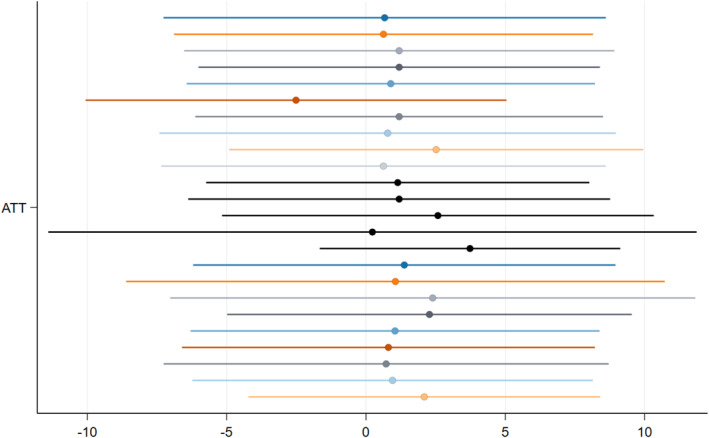
Leave‐one‐out estimates of ATT on fatalities. Each line in this plot represents the estimate and its confidence interval (using clustered standard errors) from the main specification on the deaths rate, with one state at a time excluded in alphabetical order.

**FIGURE A2:**
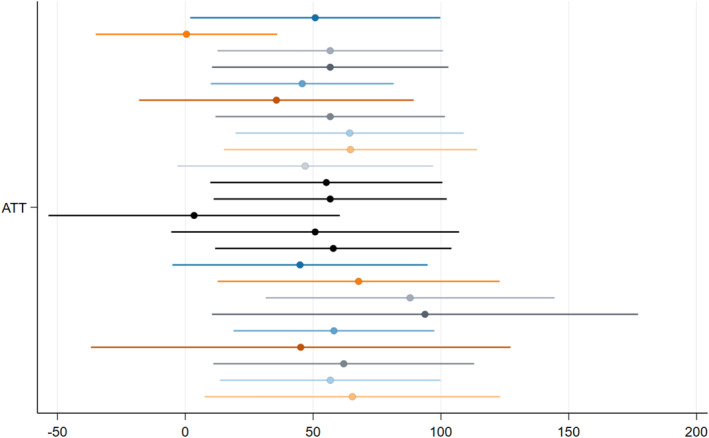
Leave‐one‐out estimates of ATT on injuries. Each line in this plot represents the estimate and its confidence interval (using clustered standard errors) from the main specification on the injuries rate, with one state at a time excluded in alphabetical order.

**FIGURE A3:**
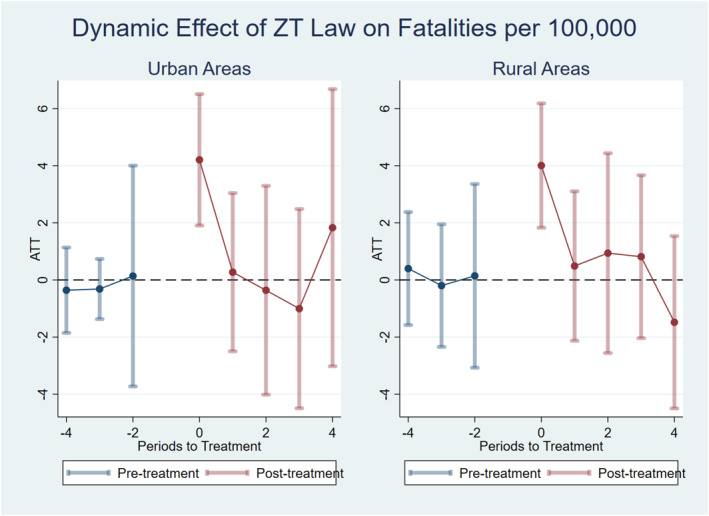
Event‐study specification for fatalities by urban status.

**FIGURE A4:**
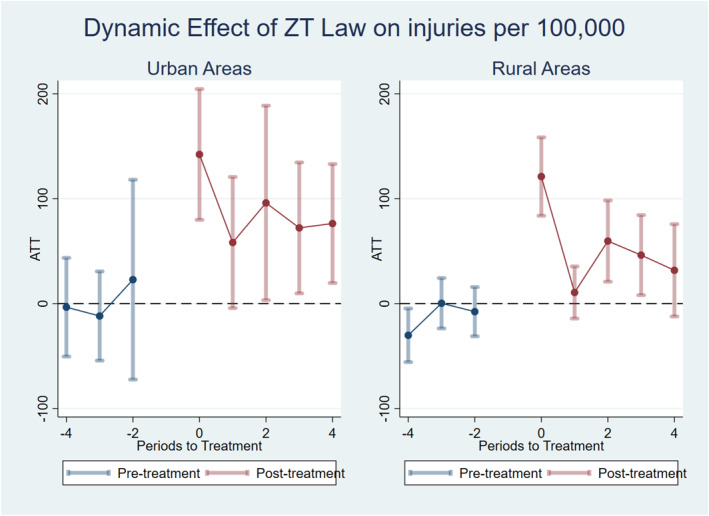
Event‐study specification for injuries by urban status.

**FIGURE A5:**
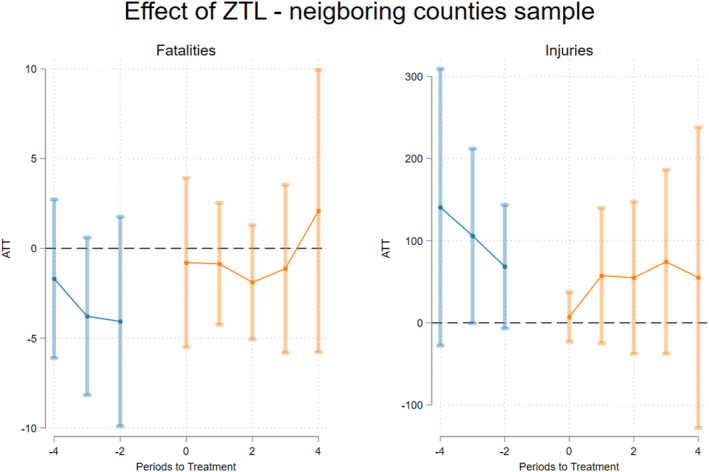
Event‐study specification for injuries: subsample of neighboring counties in different states. These event study show the estimates of an heterogeneous effects differences‐in‐differences model (Callaway and Sant’Anna 2021 including only neighboring counties in states with different policy regimes.

**FIGURE A6:**
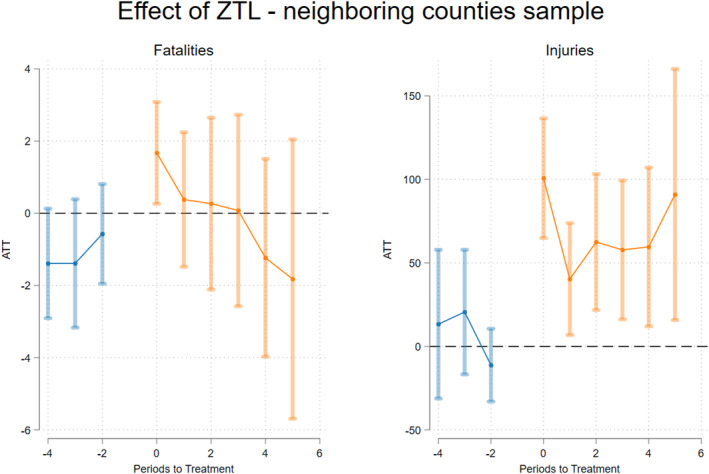
Event‐study specification for injuries: subsample excluding neighboring counties. These event study show the estimates of an heterogeneous effects differences‐in‐differences model (Callaway and Sant’Anna 2021 excluding counties neighboring an ever‐treated county.

**FIGURE A7:**
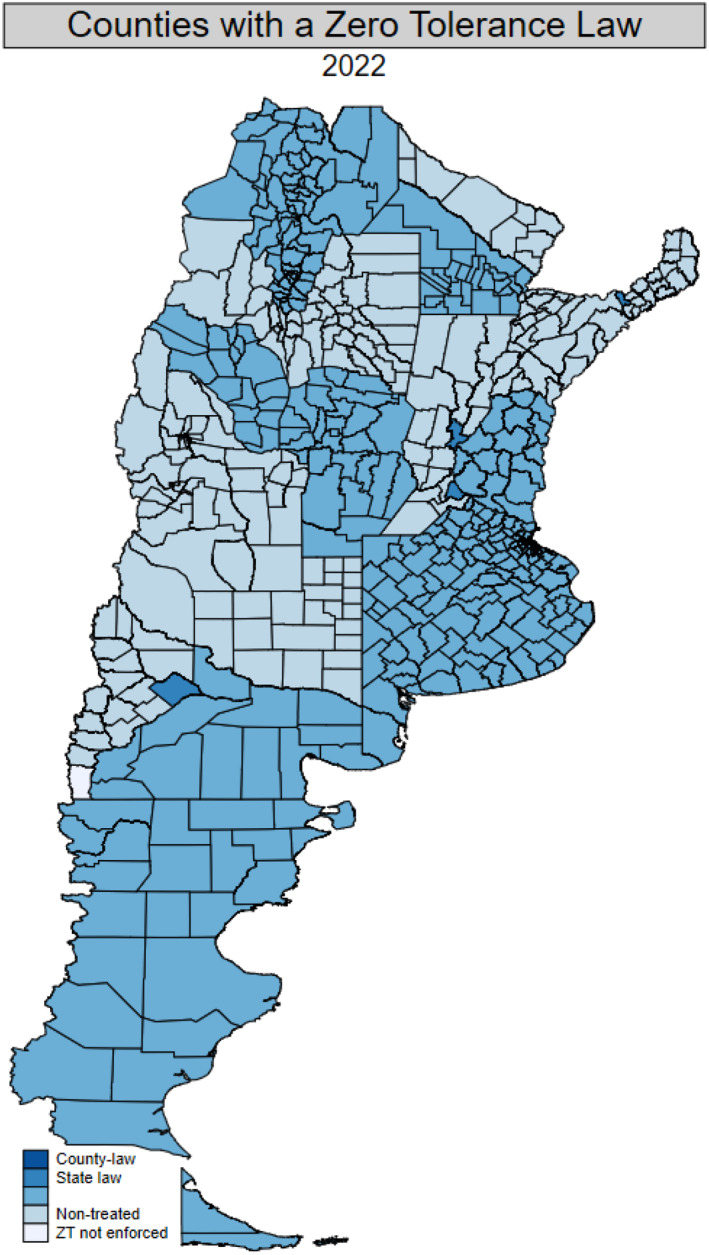
Counties with ZTLs as of 2022.

Leave‐one‐out estimates for injuries and fatalities (Appendix Figures A1 and A2) show consistent results across states. Only a few states show coefficients with wide intervals, and none show significant reversals.

#### 6.1.4 Heterogeneity by Age

Using vital statistics by age group, Table A2 confirms null effects on fatalities across all adult age brackets, consistent with overall findings. Any apparent differences may stem from sample composition rather than true heterogeneity.

## 7 Discussion

Lowering legal BAC limits has become common across Latin America. Argentina's staggered ZTL rollout since 2014, culminating in national adoption in 2023, offers a unique setting for evaluation. Using multiple data sources and DiD designs, I find that ZTLs increased injuries and had no significant effect on traffic fatalities.

This aligns with theoretical and empirical findings that suggest diminishing returns from reducing already low BAC thresholds (R. P. Compton et al. 2002; Francesconi and James 2021; Grant 2010; Grant and Lewis 2014). Mechanism tests show no effect on self‐reported alcohol use, except a drop in binge drinking. Alcohol poisoning discharges remain unaffected. This points to a weak behavioral response overall, especially among heavy drinkers, consistent with (Grant, 2016; Huang et al. 2020; Otero and Rau 2017).

Positive and significant increases in injuries may reflect perverse incentives. Moderate drinkers may consume slightly more when the penalty for any drinking becomes fixed. Table 5 supports this with increased probability of consuming over two drinks.

Overall, ZTLs appear ineffective at reducing fatalities and may worsen injury rates. These findings emphasize the need for more comprehensive drunk‐driving policies, potentially including stricter enforcement, alcohol sale regulations, and higher penalties, as shown effective elsewhere (Barron et al. 2022; Hansen 2015; Sviatschi 2008).

## Conflicts of Interest

The author declares no conflicts of interest.

## Data Availability

The data that support the findings of this study are available from the corresponding author upon reasonable request.

## Endnotes

^1^
In Argentina, road traffic fatalities represent the leading cause of accidental death and drunk driving deaths account for 30 percent of traffic fatalities (ANSV 2022).

^2^
For ease of exposition, I refer to Argentina's provinces and municipalities/departments as states and counties, respectively.

^3^
Henceforth, I refer to Callaway and Sant’Anna (2021) as CS.

^4^
In tables A4 and A3 the adoption time of ZTL for each county/municipality and state is provided.

^5^
See Table A6.

^6^
A municipality is the equivalent of a county seat.

^7^
See (ANSV 2022; Guerrera, n.d.) for reference.

^8^
ONSV was a dependent body of the ANSV.

^9^
A proxy for labor formality.

^10^
I use 2013 as the pretreatment year as this was the year before the first state to implement a ZTLdid so.

^11^
I follow Callaway and Sant’Anna (2021) and denote comparison groups accordingly.

^12^
I use private employment as a proxy for formal employment.

^13^
Given the two‐period nature of the data, TWFE does not suffer from negative weighting concerns (Goodman‐Bacon 2021).

## Appendix

**TABLE A1:** Difference‐in‐differences estimates on fatalities with ANSV and MS data.

	(1)	(2)	(3)	(4)
	Fatalities	Fatalities	Fatalities	Fatalities
	ANSV	MS	ANSV	MS
ZTL	−0.17	−0.526	−0.126	0.597
	(0.10)	(0.72)	(0.09)	(0.654)
Province FE	Yes	Yes	Yes	Yes
Year FE	Yes	Yes	Yes	Yes
Observations	2179	408	1944	384
Frequency	Monthly	Annual	Monthly	Annual
Mean of Dep. Variable	0.543	10.16	0.557	9.99
Excluding 2020	No	No	Yes	Yes

*Note:* Standard errors in parentheses. Observations weighted by population.

*p<0.10.

**p<0.05.

***p<0.01.

**TABLE A2:** Differences‐in‐differences estimates for fatalities per 100,000 people (by age group).

		Age			
	Full Sample	16–25	26–35	36–45	46+
ZTL	−0.61	−1.27	−2.55	0.52	−0.53
	(1.81)	(7.96)	(8.69)	(1.87)	(1.2)
N	2630	380	382	375	1493
Mean of Dep. Variable	10.9	18.8	17.7	11.9	6.28
State FE	Y	Y	Y	Y	Y
Year FE	Y	Y	Y	Y	Y

*Note:* Outcome variable is traffic fatalities per 100,000 people. Wild bootstrap standard errors clustered at state level are reported in parentheses. Each cell in this specification is an age group in a state in a given year. */**/*** indicates significance at the 10/5/1% levels.

*Source:* National vital statistics.

**TABLE A3:** Adoption times for states that passed ZTLs.

State‐level law	
State	Year
Buenos Aires	2022
Chaco	2022
Chubut	2020
Córdoba	2014
Entre Ríos	2018
Jujuy	2019
La Pampa	2022
La Rioja	2022
Río Negro	2017
Salta	2015
Santa Cruz	2018
Tierra del Fuego	2022
Tucumán	2016

**TABLE A4:** Adoption times for counties that passed ZTLs.

County‐level law	
City/County	Year
Mar del Plata, PBA	2018
Ezeiza, PBA	2021
Tigre, PBA	2021
Moreno, PBA	2020
Bragado, PBA	2021
Posadas, MIS	2016
Neuquén, NQN	2016
Rosario, SFE	2021
Santa fe, SFE	2020
Ushuaia, TDF	2018
Río Grande, TDF	2018
Viedma, RNE	2020
Morón, PBA	2022

**TABLE A5:** LPM estimates of ZTLs for behavioral outcomes.

	(1)	(2)	(3)	(4)
	Drinking	Drinking	Binge	Drunk
		habit	drinking	driving
ZTL x POST	0.0134	0.00622	−0.0000418	−0.0139
	(0.0242)	(0.0187)	(0.0212)	(0.0147)
Weighted	No	No	No	No
State FE	Yes	Yes	Yes	Yes
Year FE	Yes	Yes	Yes	Yes
Observations	49,175	26,033	30,389	30,644
Mean of Dep. Var.	0.630	0.156	0.147	0.215

*Note:* Standard errors in parentheses. Standard errors clustered at the state level in parentheses. Dependent variables are binary and take value 1 when the interviewed individual answers affirmatively to the questions. Coefficients reported are average marginal effects from a probit model. Column 1 includes observation for the entire sample who responded to the survey. columns 2 and three exclude the observations for individuals who do not drink and differ only in their nonresponse rate. Column 4 excludes the observations for individuals who do not drive in a regular basis.

*Source:* National Risk Factor Survey.

*p<0.10.

**p<0.05.

***p<0.01.

**TABLE A6:** Alcohol‐related fines and penalties by province.

Province	0.0–0.2 g/L	0.2–0.5 g/L	0.5–0.75 g/L	0.75–1.0 g/L	1.0+ g/l
Río Grande (TDF)	750 UP fine	1500 UP fine + 30 days disqualification	3000 UP fine + 90 days disqualification	3000 UP fine + 120 days disqualification	—
Tucumán	100‐200 UF fine + license retention until payment + possible vehicle confiscation	201‐400 UF fine + license retention until end of disqualification (2–6 months) + possible vehicle confiscation	401‐700 UF fine + license retention until end of disqualification (6 months ‐ 1 year) + vehicle retention	701–1000 UF fine + license retention until end of disqualification (1–2 years) + vehicle retention	Maximum fine and disqualification if it involves passenger or cargo transport
La Rioja	—	Up to 30 days of arrest + fine + possible vehicle retention	200 UF fine (0.5–1.0 g/L)	400 UF fine (1.0–1.001 g/L)	800 UF fine (1.001+ g/l) + up to 180 days disqualification if a repeat offender
Córdoba	Up to 100 UF fine (minor offenses)	Up to 200 UF fine (serious offenses)	Up to 400 UF fine (very serious offenses)	Up to 400 UF fine (very serious offenses)	Up to 2000 UF fine for unauthorized driving, with penalties for serious violations
Salta	100 UF fine + license retention until payment + possible vehicle confiscation	200‐400 UF fine + license retention until end of disqualification (2–3 months) + possible vehicle confiscation	401‐600 UF fine + license retention until end of disqualification (6 months) + vehicle retention	—	1000 UF fine + up to 2 years disqualification + vehicle retention
